# Sweet Woodruff (*Galium odoratum* L.)—More than Just Coumarin: From Fundamental to Biomass Valorization

**DOI:** 10.3390/molecules31162920

**Published:** 2026-08-21

**Authors:** Isabelle Herre, Thomas Stegemann

**Affiliations:** Pharmazeutisches Institut, Abteilung Pharmazeutische Biologie, Christian-Albrechts-Universität zu Kiel, Gutenbergstraße 76, 24118 Kiel, Germany; iherre@pharmazie.uni-kiel.de

**Keywords:** *Galium odoratum*, phytochemistry, coumarin, melilotosides, iridoid glycosides, analytical methods, post-harvest metabolism, toxicology, biomass valorization, circular bioeconomy

## Abstract

*Galium odoratum* (L.) Scop., commonly known as sweet woodruff, is a perennial European woodland herb traditionally used as a medicinal and aromatic plant. Its characteristic sweet, hay-like scent is mainly linked to coumarin, which is formed from glycosidically bound melilotoside precursors during wilting and drying. However, the phytochemistry of *G. odoratum* extends beyond coumarin. The species also contains iridoid glycosides, coumarin-related melilotosides, caffeoylquinic acids, flavonoids, and volatile constituents. This review summarizes current knowledge on traditional use, phytochemical constituents, metabolite dynamics, biological and ecological relevance, toxicology, and potential applications. Seasonal variation, organ-specific differences, post-harvest metabolism, and differences in analytical methodology affect analytical interpretation of the available data. Reported biological activities are mainly based on isolated constituents, in vitro studies, animal models, or non-standardized extracts, and no convincing clinical evidence is available for defined *G. odoratum* preparations. Potential applications require standardized plant material, controlled processing, sustainable sourcing, and safety assessment, while the valorization of processing residues may provide an additional route toward more resource-efficient utilization of the species. Overall, *G. odoratum* should be regarded as a chemically diverse species rather than merely as a source of coumarin aroma.

## 1. Origin and Botanical Systematics of Sweet Woodruff

*Galium odoratum* (L.) Scop. (syn. *Asperula odorata* L.), commonly known as sweet woodruff, is a perennial herb of the family Rubiaceae, subfamily Rubioideae, native across temperate Eurasia and parts of North Africa [1]. Within Europe, it is mainly associated with deciduous and mixed forests. It grows on moist, nutrient-rich, and humus-rich soils in semi-shaded to shaded sites and can form dense stands through vegetative spread [2].

**Figure 1:**
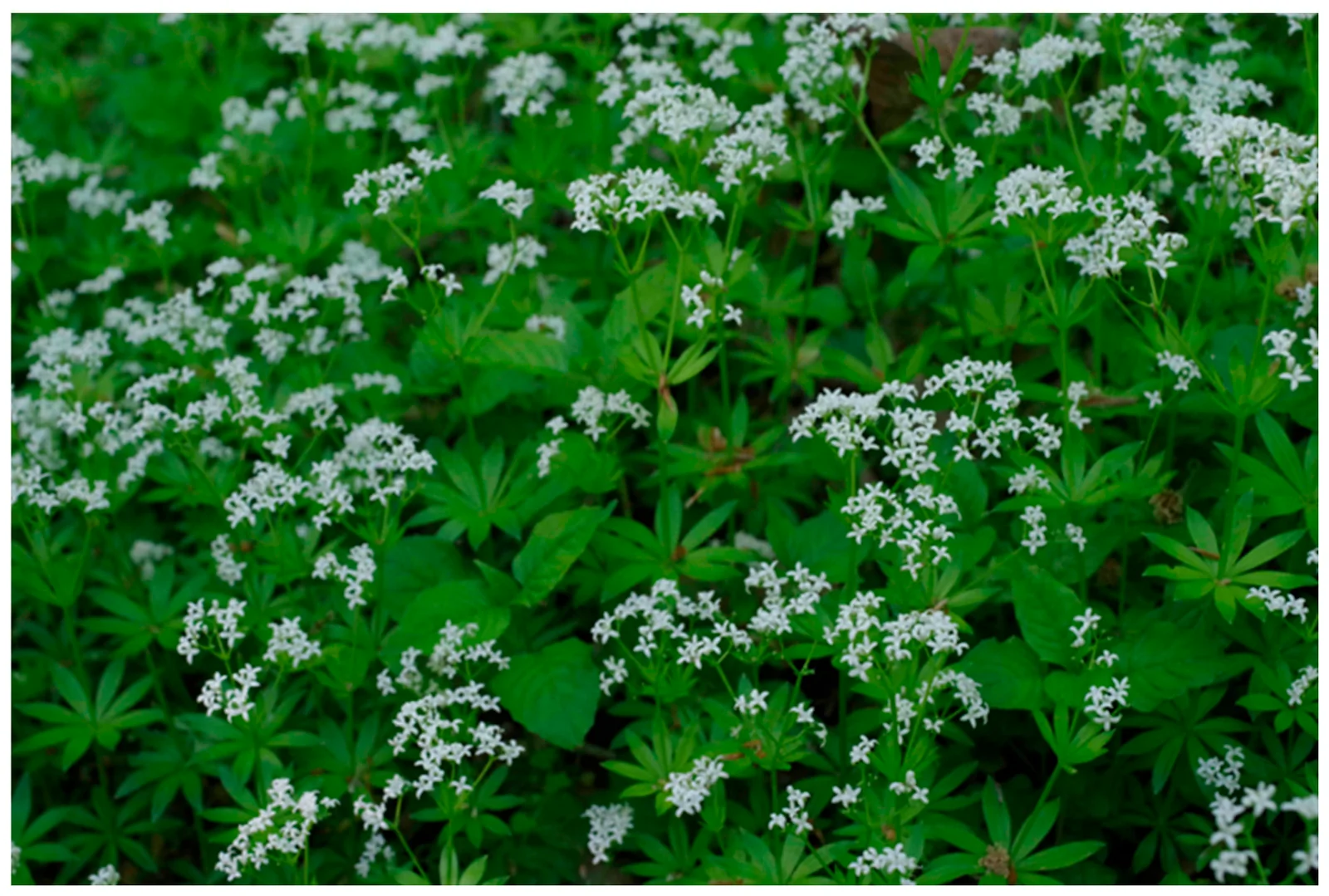
*Galium odoratum* (L.) Scop. in its natural habitat.

Morphologically, *G. odoratum* is characterized by slender, quadrangular stems, whorled narrow-lanceolate leaves, and small white flowers that appear in spring and early summer (Figure 1) [2].

This review summarizes current knowledge on traditional use, phytochemistry, metabolite dynamics, biological and ecological significance, toxicology, and potential applications of *G. odoratum*. It highlights the chemical diversity of sweet woodruff beyond coumarin.

## 2. Sweet Woodruff as a Natural Resource

### 2.1. Traditional and Contemporary Uses

*Galium odoratum* has a long history of use in Central and Northern Europe as a medicinal and aromatic plant (MAP). Traditionally, the aerial parts have been used for flavoring beverages and food preparations, particularly Maibowle or Maiwein. According to Madaus [3], the use of sweet woodruff in Maiwein can be traced back to 854 AD; he also noted that the coumarin odor increases during wilting. Dried plant material has also been used in aromatic household preparations and herbal mixtures.

In folk medicine, *G. odoratum* was used externally to treat wounds and internally to treat digestive disorders, coughs, and anxiety [3].

Today, sweet woodruff remains relevant primarily as an aroma-providing plant in beverages, desserts, confectionery, herbal preparations, and fragrance-related products. In German-speaking countries, woodruff flavor is also associated with syrups, soft drinks, and beer-based beverages such as Berliner Weisse with woodruff syrup.

### 2.2. Phytochemical Constituents

#### 2.2.1. Iridoids

**Figure 2:**
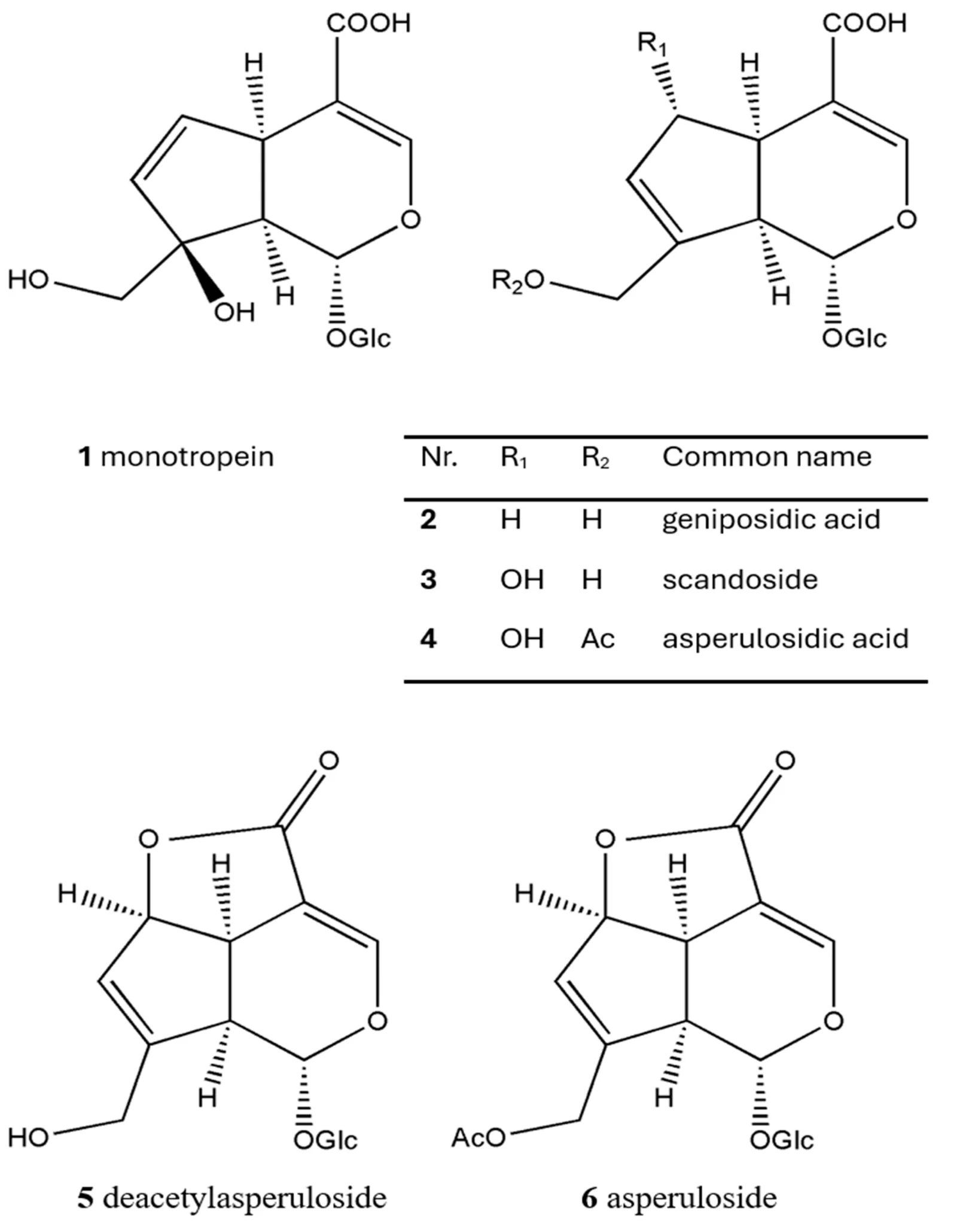
Iridoids identified in *Galium odoratum*.

*G. odoratum* contains several iridoid glycosides, including monotropein **1**, geniposidic acid **2**, scandoside **3**, asperulosidic acid **4**, deacetylasperuloside **5**, and asperuloside **6** (Figure 2) [4,5]. Iridoid glycosides are common in Rubioideae and are therefore relevant from a chemotaxonomic perspective. Several of these iridoids have also been investigated for anti-inflammatory, cytoprotective, and defensive properties, as discussed in Section 2.4.

#### 2.2.2. Phenylpropanoid-Derived Compounds: Coumarin, Melilotosides, and Caffeoylquinic Acids

**Figure 3:**
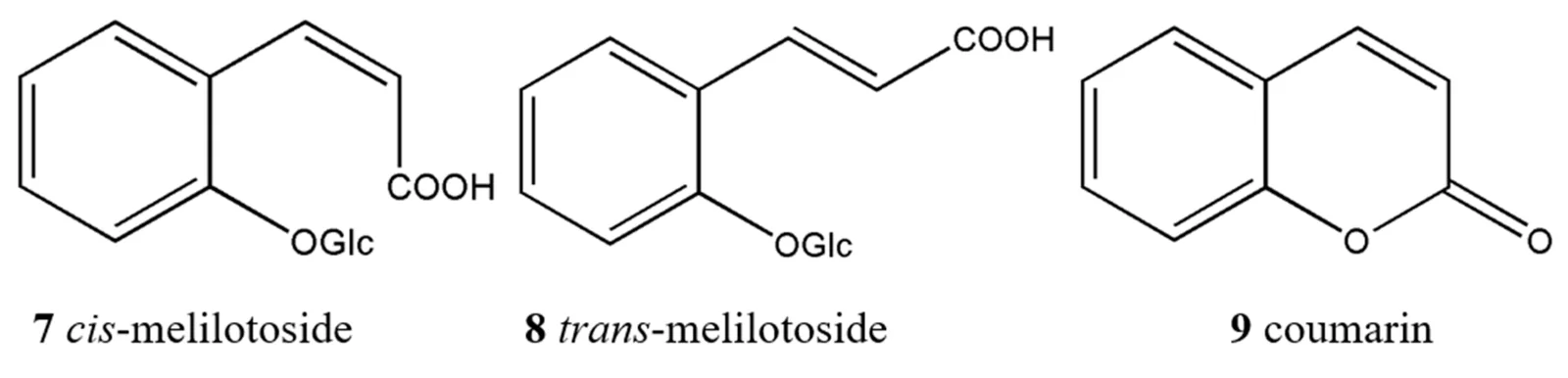
Coumarin and its precursors identified in *Galium odoratum*.

Coumarin **9** is one of the most characteristic specialized metabolites of *G. odoratum* and is primarily responsible for the sweet, hay-like aroma of sweet woodruff. It is a phenylpropanoid-derived benzopyrone. In fresh plants, coumarin **9** occurs mainly in glycosidically bound precursor forms, particularly as *cis*-melilotoside **7** and *trans*-melilotoside **8**, which can be converted into free coumarin **9** during post-harvest processing (Figure 3). This explains why aroma development depends strongly on wilting, drying, and tissue disruption, as discussed in Section 2.3.3.

**Figure 4:**
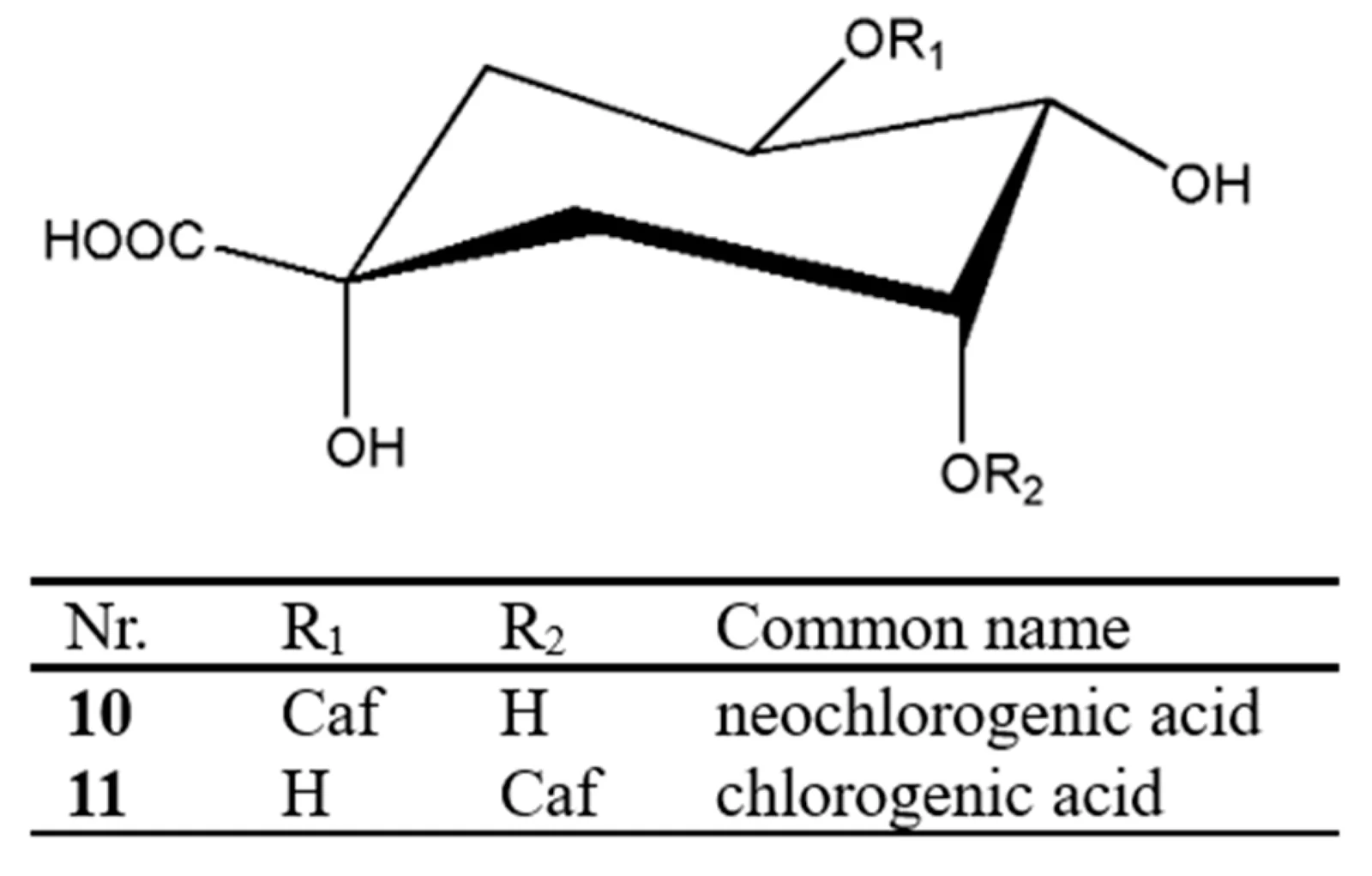
Chlorogenic acid derivatives identified in *Galium odoratum*.

Further phenylpropanoid-derived metabolites reported from *G. odoratum* include neochlorogenic acid **10** and chlorogenic acid **11** (Figure 4). Comparative studies of Romanian *Galium* species also quantified chlorogenic acid **11** in aerial parts of *G. odoratum* [6]. These caffeoylquinic acids broaden the phenylpropanoid profile beyond the coumarin-melilotoside system.

#### 2.2.3. Flavonoids

**Figure 5:**
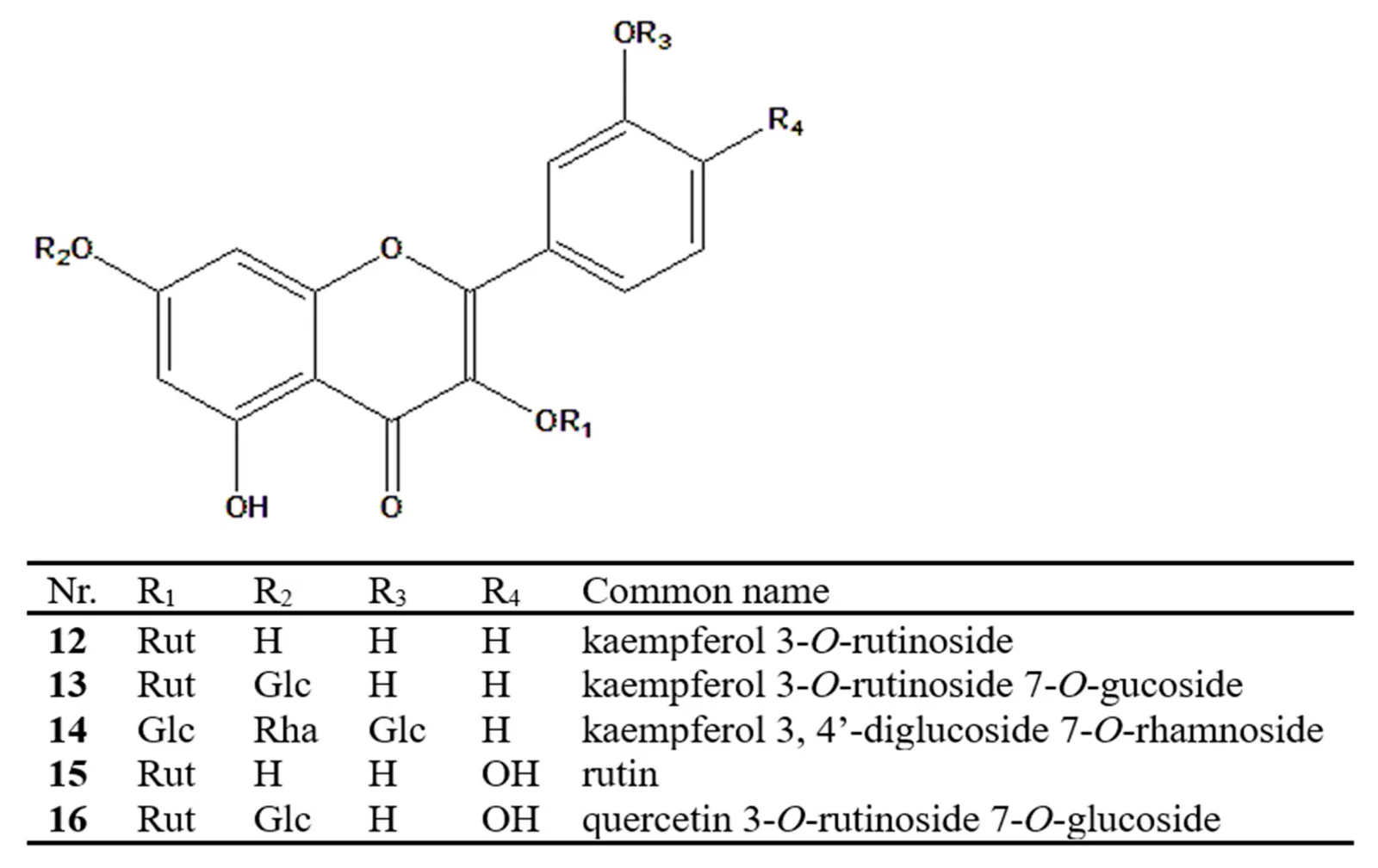
Flavonoids identified in *Galium odoratum*.

Flavonoids represent another relevant group of specialized metabolites in *G. odoratum*. Reported compounds include kaempferol 3-O-rutinoside **12**, kaempferol 3-O-[α-L-rhamnosyl-(1→6)-β-D-glucosyl]-7-O-β-D-glucoside **13,** kaempferol 3,4′-diglucoside-7-rhamnoside **14**, rutin **15**, and quercetin 3-*O*-rutinoside-7-*O*-glucoside **16** (Figure 5) [5,7]. Earlier comparative work also reported rutin, quercitrin, quercetin, and kaempferol in ethanolic extracts of *G. odoratum* aerial parts [8]. Overall, the available data indicate that *G. odoratum* contains mainly quercetin- and kaempferol-derived flavonoids. Compound numbering and the order of presentation in Figure 5 follow the sequence of first discussion in the text.

#### 2.2.4. Volatile Constituents and Essential Oil

Volatile constituents have also been reported from *G. odoratum*. Although sweet woodruff is not a typical essential-oil plant, hydrodistillation of dried aerial parts from Turkish material yielded only a low amount of essential oil (0.05%). In this oil, 96 compounds were identified, representing 89.1% of the oil. The major constituents were thymol and isothymol, followed by smaller amounts of isothymyl butyrate, linalool, α-humulene, caryophyllene oxide, and other volatile compounds [9]. Thus, the reported oil has a phenolic monoterpene-rich profile. *G. odoratum* contains volatile constituents, but its typical aroma remains mainly linked to post-harvest coumarin formation. The species is therefore better regarded as an aromatic plant, not as a typical essential-oil plant.

#### 2.2.5. Analytical Approaches, Identification Reliability, and Coverage of Reported Constituents

The phytochemical literature on *G. odoratum* spans classical isolation studies, chromatographic fingerprinting, targeted quantification, and modern mass-spectrometric profiling (Table 1). Iridoids and selected flavonoid glycosides have been isolated and characterized by spectroscopic methods, whereas comparative surveys have also used thin-layer chromatography, HPLC, UHPLC-DAD, and LC-MS-based approaches [4,5,6,7,8]. Coumarin and melilotosides have been quantified by UHPLC-DAD in fresh and drying plant material and in seasonal series [5,10]. Volatile profiles were obtained after hydrodistillation and gas-chromatographic analysis [9].

Differences in plant organ, developmental stage, post-harvest treatment, extraction solvent, chromatographic platform, and identification criteria limit direct comparison among studies. Constituents confirmed by isolation, NMR, or authentic standards should be distinguished from compounds assigned mainly from retention behavior, mass spectra, or library matching. Within the scope of the present search, all structurally characterized non-volatile constituents identified in eligible publications were included. For the volatile oil, the main text summarizes the major components; the complete list of 96 reported entries remains available in the primary GC study [9].

### 2.3. Phytochemical Distribution and Seasonality

The phytochemical composition of *G. odoratum* depends on plant organ, developmental stage, harvest time, and post-harvest handling. Such variation is common in perennial medicinal and aromatic plants and reflects developmental and environmental influences on specialized metabolism [10,14,15]. In *G. odoratum*, these factors affect aroma formation, ecological interpretation, and coumarin-related safety.

#### 2.3.1. Organ-Specific Distribution

The phytochemical profile of *G. odoratum* depends on the plant material analyzed. For traditional use and herbal raw material, the relevant material is usually the above-ground herb, including leaves and flowering shoots. Some phytochemical studies have analyzed either leaves specifically or more broadly defined aerial parts or flowering shoots. These materials should not be treated as fully equivalent.

A clearer morphological distinction exists between above-ground material and below-ground organs. Roots and rhizomes are much less well studied. Older studies reported anthraquinone pigments and related naphthalenic compounds from roots or rhizomes of *Galium* species and *Asperula odorata* [12,13], but modern phytochemical data for below-ground organs of *G. odoratum* remain scarce. Comparisons between herbal and root material should therefore be made with caution.

Clear definition of the analyzed material is especially important for coumarin-related metabolites, iridoids, phenolic acids, and flavonoids.

#### 2.3.2. Seasonal Variation

Seasonal variation in specialized metabolites is common in perennial MAPs and is linked to phenology, environmental conditions, and biotic interactions [10,14,15,16,17]. For *G. odoratum*, species-specific data have long been limited. Earlier studies mainly addressed post-harvest coumarin formation, geographic or population-related variation, and environmental effects such as growth conditions or sunlight exposure [5,7,18]. A recent two-year study investigated seasonal changes in coumarin, *cis*- and *trans*-melilotoside, and chlorogenic acid in air-dried leaves of *G. odoratum*. Concentrations declined from late April to July and increased again in August, especially for *cis*-melilotoside. Coumarin and melilotosides were generally higher in 2023 than in 2022 [11].

The traditional use of sweet woodruff in May is phytochemically plausible, because this harvest period coincides with an early-season phase in which coumarin-related metabolites are still prominent. However, available seasonal data do not identify May as a generally optimal harvest date, because metabolite levels vary between years and may increase again later in the season.

These patterns are particularly relevant for the coumarin-melilotoside system. The stronger fluctuations of melilotosides compared with free coumarin support their role as the major conjugated reservoir of coumarin-related metabolites in planta. In dried material, measured coumarin levels therefore reflect both the metabolite pool at harvest and post-harvest transformations.

Harvest time may influence precursor composition, aroma potential, phenylpropanoid profile, and toxicological interpretation. Phytochemical studies on *G. odoratum* should therefore document harvest date, developmental stage, plant organ, and post-harvest treatment.

#### 2.3.3. Post-Harvest Metabolism

**Figure 6:**
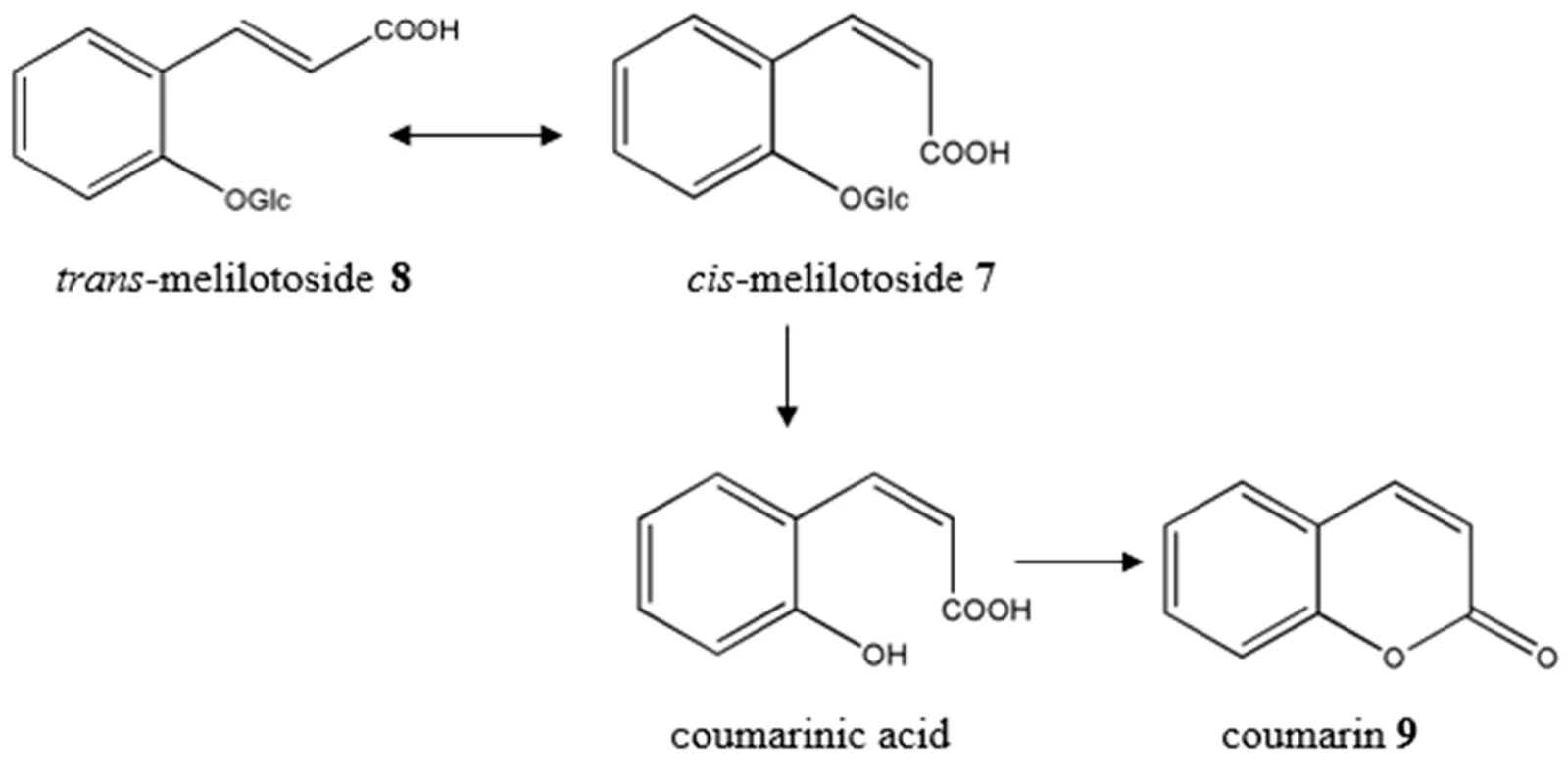
Proposed post-harvest conversion of *cis*- and *trans*-melilotoside into free coumarin in *Galium odoratum* during wilting and drying. Adapted from [5].

The phytochemical profile of *G. odoratum* changes substantially after harvest, especially within the coumarin system. During wilting, drying, or mechanical disruption, cellular compartmentation is lost, and enzymatic reactions are initiated. Glycosidases can hydrolyze *cis*- and *trans*-melilotoside, releasing aglycones that subsequently undergo lactonization to form free coumarin (Figure 6), as shown for *G. odoratum* [5]. This explains why the typical aroma becomes more pronounced after wilting or drying.

From an analytical perspective, measured coumarin levels may depend on sample handling, drying duration and conditions, extraction procedure, and storage. Studies on *G. odoratum* should therefore distinguish clearly between fresh, wilted, dried, and stored plant material.

### 2.4. Biological and Ecological Significance of Specialized Metabolites

#### 2.4.1. Pharmacological Activities Reported for Extracts and Isolated Metabolites

##### Anti-Inflammatory Activity

Anti-inflammatory activity has been reported at the extract level for *G. odoratum*. In a screening study of Italian medicinal plants, an ethanol extract of *G. odoratum* leaves inhibited carrageenin-induced paw edema in rats by 25% after oral administration at 100 mg/kg [19]. A more recent study of 70% ethanolic extracts from wild and cultivated material reported effects on polarization of murine bone marrow-derived macrophages and linked extract activity to chemically characterized phenolic profiles [7]. These findings provide direct but still preclinical extract-level evidence; they do not establish efficacy of a standardized herbal preparation in humans.

Anti-inflammatory effects have also been reported for iridoids occurring in *G. odoratum*, especially monotropein **1**, asperulosidic acid **4**, and asperuloside **6**. These compounds reduced inflammatory mediators such as nitric oxide, prostaglandin E2, TNF-α, IL-1β, and IL-6 in experimental models, mainly through NF-κB- and MAPK-associated pathways [20,21,22,23]. These data support the anti-inflammatory relevance of this compound class, but do not establish the activity of defined *G. odoratum* preparations.

##### Anti-Oxidative and Cytoprotective Effects

At the extract level, antioxidant activity has also been reported for *G. odoratum*. Methanolic and aqueous extracts showed DPPH radical-scavenging activity, with the aqueous extract being more active [24]. In the same study, topical extract formulations improved wound contraction and histological parameters in a rat second-degree burn model, but the active constituents were not identified. Phenolic-rich extracts characterized by UHPLC-MS methods also showed antioxidant activity that varied with cultivation and light exposure [7].

Several iridoids reported from *G. odoratum*, especially monotropein **1**, have also been associated with anti-oxidative and cytoprotective activities. Reported effects included reduced oxidative stress and modulation of Nrf2/HO-1- and Akt/mTOR-associated signaling pathways [23,25,26]. Cytoprotective and nephroprotective effects were reported in osteoblast, endothelial, and renal injury models, including cisplatin-induced acute kidney injury [23]. However, these data come from isolated compounds and experimental models. Their relevance for *G. odoratum* preparations remains unclear, because the concentrations or doses used in pharmacological assays cannot currently be related to quantified monotropein levels in *G. odoratum* plant material or extracts.

##### Osteoprotective and Anti-Nociceptive Effects

Monotropein **1** has also been reported to exhibit osteoprotective and anti-nociceptive properties. Experimental studies showed inhibition of osteoclast differentiation, promotion of osteoblast proliferation and mineralization, and improved bone microarchitecture in ovariectomized animal models. These effects were associated with regulation of RANKL/NFATC1- and NF-κB-related signaling pathways [20,23,27]. In rodent models, monotropein **1** also reduced writhing responses and inflammatory pain behavior, indicating anti-nociceptive activity linked to suppression of inflammatory mediators [23,28]. However, these studies were performed with isolated monotropein **1** and do not establish osteoprotective or anti-nociceptive effects of *G. odoratum* preparations.

##### Antiprotozoal and Antimalarial Activity

Phenylpropanoid-derived compounds from *G. odoratum* have also been associated with antiparasitic effects. Melilotoside has been reported to show in vitro activity against the protozoan pathogens *Entamoeba histolytica* and *Giardia lamblia* [29], whereas *trans*-melilotoside isolated from *Ajuga laxmannii* showed moderate in vitro activity against *Plasmodium falciparum* [30]. These data suggest that melilotosides may have biological relevance beyond their role as coumarin precursors. However, these effects derive from experimental systems and from compounds isolated from other plant species, and their relevance for *G. odoratum* preparations or traditional uses remains unclear.

##### Coumarin and the Wider Coumarin Class

Coumarin **9** should be distinguished from anticoagulant 4-hydroxycoumarin derivatives and from the large, structurally diverse group of natural and synthetic coumarins used in medicinal-chemistry studies. Across this wider class, anti-inflammatory, antioxidant, antimicrobial, anticancer, enzyme-modulating, and other activities have been reported, predominantly in vitro or in animal models [31]. This broad literature demonstrates the pharmacological versatility of the coumarin scaffold but does not establish that simple coumarin, at concentrations occurring in *G. odoratum* preparations, produces a defined therapeutic effect. General coumarin pharmacology should therefore not be presented as direct evidence for sweet woodruff, particularly because coumarin exposure is constrained by toxicological and regulatory considerations (Section 2.5).

**Figure 7:**
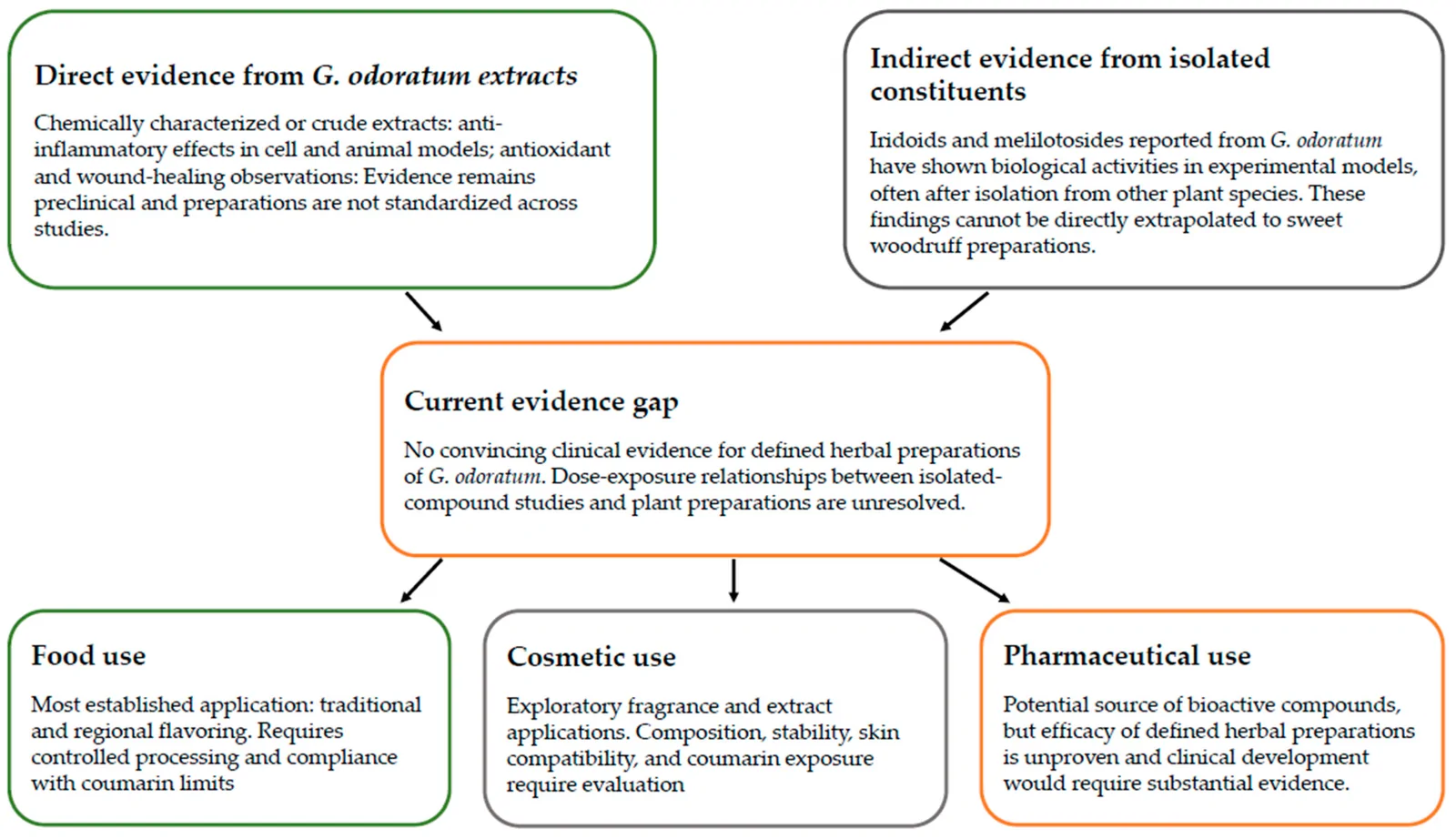
Evidence for biological effects of *Galium odoratum* and its relevance for food, cosmetic, and pharmaceutical applications. Colors indicate the level and type of available evidence: green denotes direct evidence or established use, gray indirect evidence or exploratory applications, and orange major evidence gaps or applications requiring further validation.

Table 2 summarizes the distinction between direct evidence from *G. odoratum* extracts and indirect evidence derived from isolated constituents, related species, or computational models. The relationship between the available biological evidence and its potential relevance for food, cosmetic, and pharmaceutical applications is summarized in Figure 7.

#### 2.4.2. Ecological Functions

##### Defense Against Herbivores and Insects

Iridoids from *Galium* species may fulfill ecological defensive functions. In *G. melanantherum*, several iridoids showed insecticidal activity against *Kalotermes flavicollis* termites and *Crematogaster scutellaris* ants [33]. These data support a possible defensive role of iridoids in *Galium*, but direct evidence for such a function in *G. odoratum* is lacking.

##### Oxidative Stress Protection and UV Screening

Flavonoids are widely recognized as plant metabolites involved in oxidative stress protection and photoprotection, including UV screening. In *G. odoratum*, quercetin and kaempferol derivatives have been reported, and antioxidant activity has been demonstrated at the extract level [7]. However, direct functional evidence for the contribution of individual flavonoids to oxidative stress protection or UV screening in this species remains limited. Their ecological relevance in *G. odoratum* should therefore be inferred cautiously from general functions in plants.

##### Aroma Formation and Ecological Interactions

As discussed in Section 2.3.3, the characteristic aroma of *G. odoratum* is mainly linked to post-harvest coumarin formation. This process is most relevant for human perception and traditional use. In the plant, coumarin-related and other phenylpropanoid-derived compounds may also contribute to ecological interactions, for example as chemical cues or as part of defense responses. Coumarin has been identified as a biting-deterrent constituent in the traditional mosquito-repellent plant *Hierochloë odorata* [34]. However, direct evidence for a comparable repellent function in *G. odoratum* is lacking.

More broadly, the specialized metabolite profile of *G. odoratum* may reflect adaptation to shaded woodland habitats. Possible ecological functions include defense and stress protection, but direct evidence remains limited.

Overall, evidence for the biological activity of whole-plant preparations of *G. odoratum* remains limited. Most reported effects derive from isolated constituents, in vitro assays, animal models, or non-standardized extracts. No convincing clinical evidence is currently available for defined herbal preparations of *G. odoratum*, and pharmacological findings obtained with isolated compounds should not be directly extrapolated to sweet woodruff preparations.

### 2.5. Toxicological and Regulatory Aspects

The toxicological assessment of *G. odoratum* is mainly determined by its coumarin content. Coumarin contributes to the characteristic aroma of sweet woodruff, but it is also the compound of primary regulatory relevance. Thus, the same compound that defines its traditional use also determines the main safety considerations.

#### Coumarin Intake, Hepatotoxicity, and Food Regulation

Coumarin is toxicologically relevant because liver toxicity has been identified as the critical endpoint in risk assessment. EFSA established a tolerable daily intake (TDI) of 0.1 mg coumarin/kg body weight/day and confirmed this value in a later assessment [35]. The TDI refers to regular long-term exposure and should not be interpreted as a threshold for acute toxicity. Individual susceptibility to coumarin-associated liver effects has been reported, although these effects are usually reversible after cessation of exposure.

In the European Union, coumarin is regulated under Regulation (EC) No 1334/2008 on “flavourings and food ingredients with flavouring properties” [36]. Coumarin may not be added as such to foods, but it can occur naturally in flavorings or food ingredients with flavoring properties. Annex III of the regulation defines maximum levels for coumarin in selected food categories. The use of *G. odoratum* as a natural flavoring source therefore depends on the intended product category, expected intake, and processing conditions.

For *G. odoratum*, toxicological interpretation is further complicated by post-harvest formation of free coumarin from glycosidically bound melilotoside precursors. Consequently, coumarin exposure cannot be evaluated independently of harvest time, plant organ, drying conditions, storage, extraction procedure, and preparation method. This is particularly relevant for beverages and foods prepared from wilted or dried plant material, where aroma formation and coumarin release are closely linked.

Overall, the toxicological relevance of *G. odoratum* does not preclude its traditional or contemporary use, but it requires controlled preparation, moderate intake, and transparent declaration of plant material and processing conditions.

### 2.6. Potential Applications, Sustainable Sourcing, and Biomass Valorization

The phytochemical profile of *G. odoratum* may support applications in the food, cosmetic, and pharmaceutical sectors. However, practical use requires standardized plant material, controlled post-harvest processing, and careful consideration of coumarin exposure.

Sourcing is also relevant. *G. odoratum* is a regional European woodland species and has been listed among wild-collected MAPs in Poland [37]. In Poland, it was formerly partly protected but has been classified as not protected since 2015. Species-specific harvest volumes are poorly documented; broader use should therefore rely on cultivated material or controlled wild collection.

Compared with non-European coumarin-rich sources such as tonka beans, *G. odoratum* may be of interest as a regional European source of woodruff-like aroma. Its potential relevance lies less in high coumarin yield than in regional sourcing and traditional use.

#### 2.6.1. Food Industry

Food use represents the most established field of application for *G. odoratum*. Sweet woodruff has long been used as an aroma-providing plant in beverages, desserts, confectionery, and seasonal preparations such as Maibowle or Maiwein. Its characteristic scent is mainly linked to post-harvest coumarin formation.

From an industrial perspective, *G. odoratum* is unlikely to be a competitive source of coumarin itself, because synthetic or standardized flavoring systems are easier to control. Its relevance may therefore be limited to regional and traditional flavoring applications.

Food-related use must comply with toxicological and regulatory requirements for coumarin. Standardization of plant material, drying conditions, and extraction procedures would therefore be necessary for reproducible and safe use as a natural flavoring source.

#### 2.6.2. Cosmetic Industry

In cosmetics, *G. odoratum* may be relevant as a fragrance-related plant material and as a plant extract containing phenolic acids, flavonoids, iridoids, and coumarin-related metabolites. Extract-level antioxidant activity and wound-healing effects have been reported in a rat burn model [24], but the active constituents were not identified. Therefore, cosmetic or skin-related applications remain exploratory and require further studies on extract composition, stability, skin compatibility, and coumarin content.

#### 2.6.3. Pharmaceutical Industry

Pharmaceutical interest in *G. odoratum* is mainly linked to iridoids and other compounds with reported bioactivity. Compounds reported from the species, including monotropein, asperulosidic acid, and asperuloside, have been associated with anti-inflammatory, cytoprotective, osteoprotective, and anti-nociceptive effects. In addition, recent in silico work explored volatile *G. odoratum* constituents as potential EGFR and HER2 ligands in the context of bladder cancer. β-Caryophyllene and β-selinene showed the highest predicted affinity to EGFR, but these findings remain computational and require experimental validation [32].

From a pharmaceutical perspective, *G. odoratum* may be considered a source of compounds with reported bioactivity, but development as a conventional herbal medicinal product appears challenging. Most evidence derives from isolated compounds, extract-level studies, animal models, or in silico approaches and does not establish clinical efficacy of defined *G. odoratum* preparations. Many potential indications would also represent non-traditional uses and would therefore require substantial pharmacological, toxicological, and clinical evidence.

Any such use would require standardized material, defined quality parameters, controlled coumarin exposure, and appropriate safety data.

#### 2.6.4. Sustainable Valorization of Processing Residues

The utilization of *G. odoratum* may extend beyond the primary recovery of aroma compounds or phytochemical extracts. Processing of aerial parts for beverages, herbal preparations, fragrance products, or botanical extracts may generate spent plant biomass in which constituents not completely removed during the initial processing step remain. Such material could therefore represent a secondary source of extractable compounds.

Species-specific evidence for sequential utilization of *G. odoratum* biomass is limited but available. Earlier studies conducted under the synonym *Asperula odorata* investigated dry extracts obtained by hot-water extraction of exhausted herbal material remaining after the sequential recovery of lipophilic and phenolic complexes [38]. The resulting secondary extract showed dose-dependent effects on macrophage transformation and phagocytic activity in vitro. These findings demonstrate that an additional extract with measurable biological activity can be obtained from previously extracted *G. odoratum* biomass. However, the chemical composition and extraction yield of this secondary extract were not characterized. The study was designed to investigate pharmacological activity rather than the technological, environmental, or economic feasibility of residue valorization.

A cascade approach could therefore combine primary utilization of the aerial parts for aroma or selected phytochemicals with subsequent characterization and, where feasible, recovery of residual extractable constituents. Candidate compound classes include iridoids, phenolic acids, flavonoids, and coumarin-related metabolites known from the aerial parts, but their occurrence and recoverability after primary processing remain to be established. Studies in other plant-processing systems indicate that phenolic and flavonoid compounds may remain in biomass after primary extraction and can be recovered by subsequent extraction steps [39]. Whether this principle is applicable to *G. odoratum* remains to be investigated systematically.

Extraction procedures for secondary recovery should be evaluated with regard to solvent and energy consumption as well as selectivity and recovery efficiency. Aqueous or aqueous-ethanolic extraction and techniques such as ultrasound- or microwave-assisted extraction may be considered as potential approaches. Their suitability for *G. odoratum*, however, has not yet been established. In addition to extraction yield, residual coumarin and melilotoside concentrations, overall extract composition, safety, regulatory requirements, solvent recovery, energy demand, and the environmental consequences of additional processing steps would require assessment.

**Figure 8:**
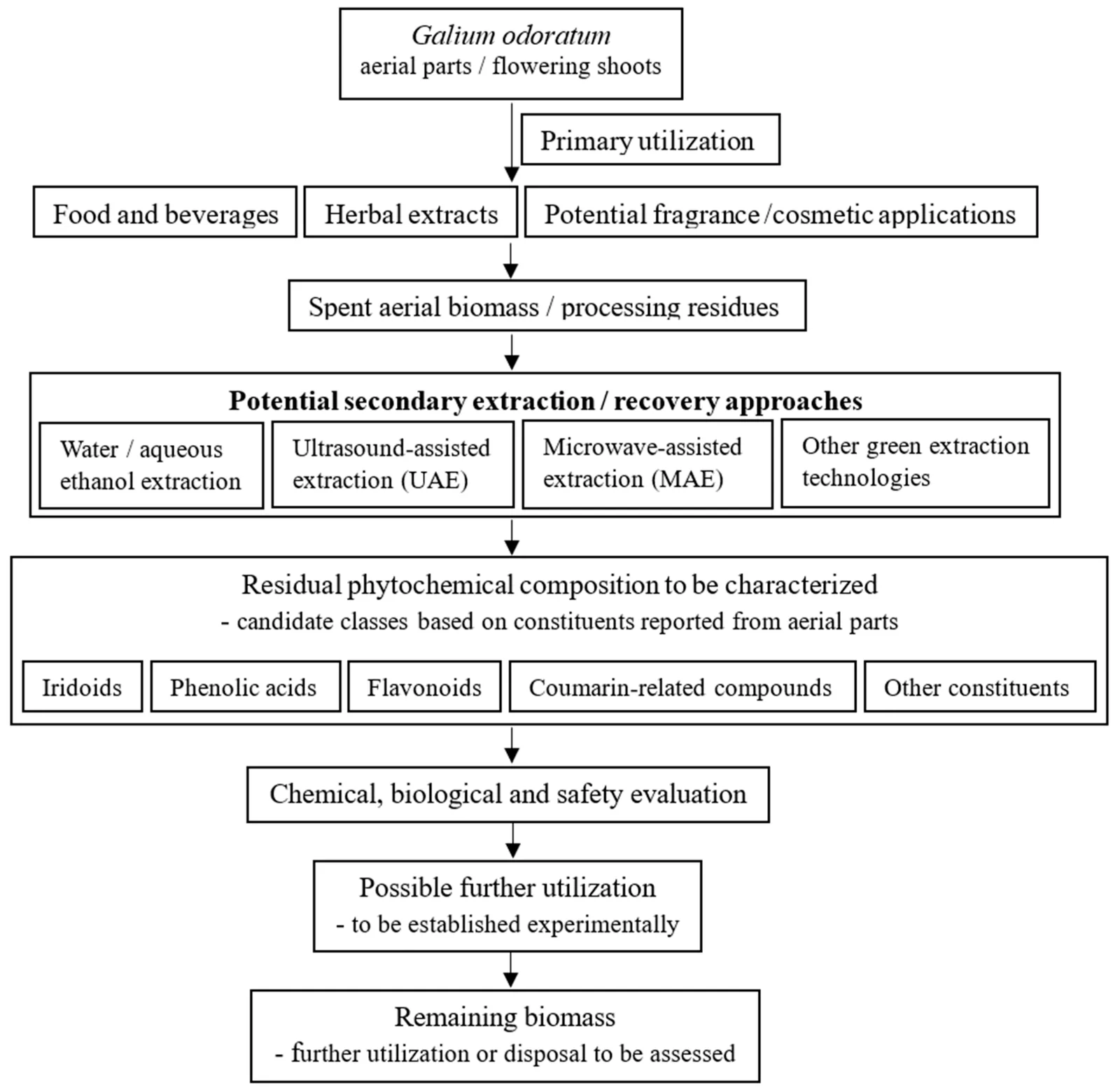
Conceptual cascade utilization of *Galium odoratum* aerial biomass. Spent biomass generated during primary utilization may be subjected to secondary extraction and characterization. Residual phytochemical composition, potential further use, and final biomass utilization remain to be established.

Within a cascade-utilization concept, sequential processing could reduce the amount of biomass remaining after primary extraction. Following secondary extraction, the remaining biomass could be evaluated for further material or biological utilization, provided that its composition and safety are sufficiently characterized. Such an approach is consistent with circular-bioeconomy and low-waste processing concepts, but its practical relevance for *G. odoratum* currently remains to be demonstrated. A conceptual cascade approach integrating primary utilization, secondary recovery of residual constituents, and subsequent biomass use is summarized in Figure 8.

Below-ground organs represent a related but distinct research area. Roots and rhizomes are not processing residues of the aerial herb commonly used for food, aromatic, or herbal applications. Older studies nevertheless reported anthraquinone pigments and related naphthalenic compounds from below-ground material [12,13], whereas modern phytochemical characterization remains limited. Systematic comparisons of aerial and below-ground organs could therefore reveal additional organ-specific constituents. However, the use of roots or rhizomes would require a separate sustainability assessment because their collection is destructive and would be particularly problematic for material obtained from wild populations.

## 3. Literature Search and Selection

This review used a narrative literature-search strategy focused on *Galium odoratum*, its synonym *Asperula odorata*, and compounds reported from the species. PubMed, SciFinder, Reaxys, Web of Science, and Google Scholar were searched up to May 2026. Core searches combined (“*Galium odoratum*” OR “*Asperula odorata*” OR “sweet woodruff”) with terms including phytochemistry, constituent, compound, coumarin, melilotoside, iridoid, flavonoid, phenolic acid, volatile, essential oil, pharmacology, biological activity, toxicology, traditional use, ecology, and application. Compound-specific follow-up searches were performed for constituents identified from *G. odoratum*, and additional records were located through reference-list screening. Additional searches combined the species names with processing residue, spent biomass, by-product, exhausted plant material, valorization, green extraction, cascade utilization, waste valorization, and circular bioeconomy.

Peer-reviewed primary studies were prioritized. Historical monographs, botanical databases, and regulatory documents were included when they supported traditional-use, taxonomic, or safety statements. Records were included when they contained traceable data on *G. odoratum* chemistry, biological activity, ecology, toxicology, or use. Studies on isolated constituents or related species were included only when they helped interpret compounds directly reported from *G. odoratum*, and such evidence was explicitly treated as indirect. Records were excluded when they concerned unrelated taxa without interpretive relevance, repeated untraceable claims, or duplicate publication of the same data. Duplicate records were consolidated manually using title, authors, year, journal, and DOI.

The search was designed to identify all structurally characterized constituents reported in the eligible literature. For complex volatile profiles, the retrieval included the complete primary reports, whereas the main narrative emphasizes major constituents and methodological limitations. The revised synthesis includes 39 publications and documents in the reference list.

## 4. Future Perspectives

Research on *G. odoratum* has long focused on coumarin and its relevance for aroma and safety. Future studies should place coumarin within the broader phytochemical profile of the species and give greater attention to iridoids, flavonoids, caffeoylquinic acids, volatile constituents, and coumarin-related melilotosides. Untargeted and targeted metabolomics could clarify this chemical diversity.

A major need is the standardization of plant material and analytical reporting. Plant organ, developmental stage, harvest date, drying conditions, storage, and extraction procedure should be clearly documented. This is essential because seasonal variation and post-harvest metabolism strongly influence the measured coumarin-melilotoside profile. Comparative analyses of fresh, wilted, dried, and stored material would help to distinguish in planta metabolite dynamics from processing-related transformation or loss.

Organ-specific studies are also needed. Most available data refer to aerial parts, leaves, or flowering shoots, whereas systematic comparisons of above-ground and below-ground organs remain limited. This would clarify the relevance of different organs for aroma, ecology, safety, and applications.

The biological relevance of *G. odoratum* remains insufficiently defined. Several activities have been reported for extracts or isolated constituents, but most evidence derives from in vitro assays or animal models. Future studies should therefore test chemically characterized and standardized preparations, establish dose-exposure relationships, assess clinical relevance where appropriate, and experimentally investigate ecological functions such as herbivore defense, photoprotection, microbial interactions, and aroma-related signaling.

Another important research direction is the valorization of processing side streams. Future studies should determine which constituents remain in *G. odoratum* biomass after infusion, aqueous or hydroalcoholic extraction, or other primary processing steps. Mass balances across sequential extraction stages would allow the recovery efficiency of individual metabolite classes to be assessed and would distinguish residual phytochemical value from compounds already depleted during primary processing. Green extraction technologies should be evaluated not only with respect to extraction yield but also solvent consumption, energy demand, selectivity, coumarin removal or enrichment, and scalability. Such studies could establish whether cascade utilization is feasible, including the secondary recovery of residual phytochemicals and subsequent evaluation of the remaining biomass for further use. In this context, zero-waste processing and circular-bioeconomy concepts provide potential frameworks, but their environmental and economic relevance for *G. odoratum* remains to be demonstrated.

Future applications of *G. odoratum* should combine phytochemical characterization with safety assessment. Food, cosmetic, or pharmaceutical applications require controlled processing, reproducible extract composition, sustainable sourcing, and careful consideration of coumarin exposure.

## 5. Conclusions

*G. odoratum* is best known as a coumarin-containing aromatic plant, but its phytochemistry extends well beyond this single compound. Iridoids, coumarin-related melilotosides, caffeoylquinic acids, flavonoids, and volatile constituents contribute to a chemically diverse profile with biological, ecological, and practical relevance. Seasonal variation, organ-specific differences, post-harvest metabolism, and analytical methodology strongly influence this profile and must be considered when interpreting analytical data, aroma development, coumarin exposure, and potential applications. Direct biological evidence for *G. odoratum* is limited to preclinical extract-level observations, whereas much of the wider pharmacological discussion concerns isolated compounds or related species. No convincing clinical evidence is available for defined herbal preparations. Sweet woodruff should therefore be regarded as a chemically diverse aromatic species whose potential applications require rigorous standardization and evidence-based safety assessment.

## Figures and Tables

**Table 1:** Overview of the principal phytochemical classes and analytical approaches reported for *Galium odoratum*.

Compound Class	Representative Constituents	Plant Material	Analytical and Identification Basis	Refs.
Iridoid glycosides	Monotropein **1**; geniposidic acid **2**; scandoside **3**; asperulosidic acid **4**; deacetylasperuloside **5**; asperuloside **6**	aerial parts; methanolic extracts	Isolation and spectroscopic characterization; TLC-densitometry and HPLC fingerprinting; comparison with authentic or reference data	[4,5]
Coumarin system	*cis*-Melilotoside **7**; *trans*-melilotoside **8**; coumarin **9**	Fresh, wilted, and dried aerial parts or leaves	Isolation and structure elucidation; quantitative UHPLC-DAD during drying and seasonal studies	[5,11]
Caffeoylquinic acids	Neochlorogenic acid **10**; chlorogenic acid **11**	Aerial parts or leaves; alcoholic extracts	Isolation and spectroscopy; HPLC/UHPLC-DAD or LC-MS-based profiling and quantification	[5,6,7,11]
Flavonoids	Compounds **12–16**; rutin; quercitrin; quercetin; kaempferol	Aqueous ethanolic or ethanolic extracts of aerial parts	Isolation/NMR for selected glycosides; UHPLC-HRMS/MS and targeted UHPLC-MS/MS; standards and literature data	[5,7,8]
Volatile constituents	96 reported constituents; major compounds include thymol and isothymol	Hydrodistilled oil from dried aerial parts	Hydrodistillation followed by gas-chromatographic profiling; results are mainly relative and method-dependent	[9]
Root/rhizome quinones	Anthraquinone pigments and related naphthalenic compounds	Roots and rhizomes	Classical isolation and pigment analysis; limited modern confirmation	[12,13]

**Table 2:** Summary and critical appraisal of pharmacological evidence relevant to *Galium odoratum*.

Evidence Category	Test Material	Model and Reported Effect	Critical Interpretation	Refs.
Direct extract evidence	*G. odoratum* ethanol or 70% ethanol extracts	Rat paw-edema model; murine macrophage polarization; reported anti-inflammatory effects	Direct species-level evidence, but preclinical and based on non-equivalent preparations	[7,19]
Direct extract evidence	Methanolic and aqueous *G. odoratum* extracts	DPPH radical scavenging and a rat burn model; antioxidant and wound-healing observations	Active constituents not established; no clinical validation	[24]
Indirect constituent evidence	Monotropein, asperulosidic acid, asperuloside	Cell and animal models; anti-inflammatory, cytoprotective, osteoprotective, and anti-nociceptive effects	Mostly isolated compounds from other source species; dose relevance to sweet woodruff is unknown	[20,21,22,23,24,25,26,27,28]
Indirect constituent evidence	Melilotosides	In vitro antiprotozoal and antimalarial assays	Compounds were studied in other plant sources or isolated systems	[29,30]
Indirect class-level evidence	Natural and synthetic coumarins	Broad preclinical activities across structurally diverse coumarins	Does not demonstrate activity of simple coumarin or *G. odoratum* preparations	[31]
Computational evidence	Volatile constituents reported from *G. odoratum*	Docking to EGFR and HER2	Hypothesis-generating only; requires experimental validation	[32]
Clinical evidence	Defined herbal preparations of *G. odoratum*	No convincing clinical studies identified	Clinical efficacy and effective dose remain unestablished	

## Data Availability

No new data were created or analyzed in this study. All sources used are cited in the reference list.

## Abbreviations

The following abbreviations are used in this manuscript:

DPPH	2,2-diphenyl-1-picrylhydrazyl
MAP	medicinal and aromatic plant
TDI	tolerable daily intake
